# Dysbiosis and Enhanced Beta-Defensin Production in Hair Follicles of Patients with Lichen Planopilaris and Frontal Fibrosing Alopecia

**DOI:** 10.3390/biomedicines9030266

**Published:** 2021-03-07

**Authors:** Andria Constantinou, Katarzyna Polak-Witka, Marios Tomazou, Anastasis Oulas, Varvara Kanti, Rolf Schwarzer, Johannes Helmuth, Anke Edelmann, Ulrike Blume-Peytavi, George M. Spyrou, Annika Vogt

**Affiliations:** 1 Charité–Universitätsmedizin Berlin, Corporate Member of Freie Universität Berlin and Humboldt-Universität zu Berlin, Clinical Research Center for Hair and Skin Science, Department of Dermatology, Venereology and Allergology, Charitéplatz 1, 10117 Berlin, Germany; andria.constantinou@charite.de (A.C.); katarzyna.polak-witka@charite.de (K.P.-W.); vera.kanti@charite.de (V.K.); ulrike.blume-peytavi@charite.de (U.B.-P.); 2 Department of Dermatology, Medical University of Warsaw, 02-008 Warsaw, Poland; 3 Bioinformatics ERA Chair, The Cyprus Institute of Neurology and Genetics & The Cyprus School of Molecular Medicine, 2371 Nicosia, Cyprus; mariost@cing.ac.cy (M.T.); anastasioso@cing.ac.cy (A.O.); georges@cing.ac.cy (G.M.S.); 4 Department Molecular Diagnostics, Labor Berlin Charité Vivantes GmbH, 13353 Berlin, Germany; rolf.schwarzer@laborberlin.com (R.S.); johannes.helmuth@laborberlin.com (J.H.); anke.edelmann@laborberlin.com (A.E.)

**Keywords:** hair follicle, scalp, alopecia, hair loss, histology, microbiome, bacteria, metagenomics, hair disease, next-generation sequencing

## Abstract

Despite their distinct clinical manifestation, frontal fibrosing alopecia (FFA) and lichen planopilaris (LPP) display similar histopathologic features. Aberrant innate immune responses to endogenous or exogenous triggers have been discussed as factors that could drive inflammatory cascades and the collapse of the stem cell niche. In this exploratory study, we investigate the bacterial composition of scalp skin and plucked hair follicles (HF) of patients with FFA, LPP and alopecia areata circumscripta (AAc), as well as healthy individuals, in relation to cellular infiltrates and the expression of defense mediators. The most abundant genus in lesional and non-lesional HFs of LPP and FFA patients was Staphylococcus, while Lawsonella dominated in healthy individuals and in AAc patients. We observed statistically significant differences in the ratio of Firmicutes to Actinobacteria between healthy scalp, lesional, and non-lesional sites of FFA and LPP patients. This marked dysbiosis in FFA and LPP in compartments close to the bulge was associated with increased HβD1 and HβD2 expression along the HFs from lesional sites, while IL-17A was increased in lesional HF from AAc patients. The data encourage further studies on how exogenous factors and molecular interactions across the HF epithelium could contribute to disease onset and propagation.

## 1. Introduction

Frontal fibrosing alopecia (FFA), an acquired primary cicatricial alopecia, affects the scalp in a characteristic clinical pattern. Previously considered a rare variant of lichen planopilaris (LPP) in postmenopausal women, it has globally emerged as a frequent diagnosis in hair clinics, also affecting younger females and males [1,2,3]. The steady rise in patient numbers and the peculiar clinical manifestations have led to an increasing interest in the underlying pathogenesis.

**Figure 1:**
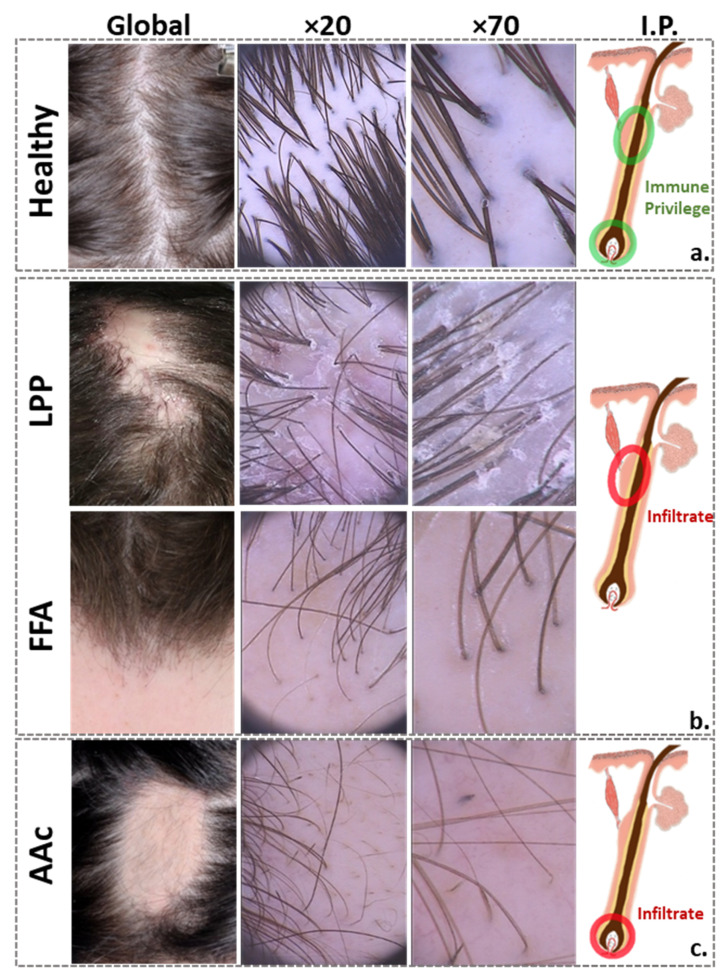
Clinical and dermoscopic pictures of healthy scalp and inflammatory scalp diseases. From left to right: representative global hair photography (VISIA-CR^®^, Canfield Scientific Inc., Fairfield, NJ, USA), dermoscopic scalp examinations at 20-fold and 70-fold magnification (Fotofinder^®^ TeachScreen Systems software GmbH; Bad Birnbach, Germany), and schematic of a HF, showing the (**a**) immune privilege areas of the healthy HF; the (**b**) inflammation sites of scarring alopecia, located around the stem cell-bearing bulge area, considered to be the “critical zone” for HF homeostasis and disease pathogenesis, because its integrity is required for stem cell survival and HF regrowth; and (**c**) non-scarring alopecia, where inflammation mainly affects the peribulbar region, allowing a possible HF regrowth. Clinically, LPP typically presents as patchy hair loss, with scaling and redness at the edges, whereas FFA typically presents with band-like, receding frontal hair line and eyebrow loss. Despite the completely different clinical characteristics of FFA and LPP, their histopathological findings are common, consisting of inflammatory lymphocytic infiltrates in the upper follicle and surrounding fibrosis, with subsequent permanent destruction of the HF. AAc usually presents with clearly defined round patches of hair loss without scarring. Histopathologically, there is a peribulbar lymphocytic infiltrate around anagen follicles and a shift towards the telogen hair growth phase. LPP, lichen planopilaris; FFA, frontal fibrosing alopecia; AAc, alopecia areata circumscripta; HF, hair follicle; I.P., immune privilege.

While the practical relevance of clinical signs of inflammation, such as perifollicular erythema and follicular casts (Figure 1) remains unclear [4], FFA and LPP share many histological features, frequently making them indistinguishable from each other [5,6]. Prominent features include a lichenoid peri-bulge infiltration and the involvement of mast cells [7]. On the cytokine level, a high presence of IFN-γ has been associated with the recruitment of immune cells via the expression of downstream chemokines, and with the epithelial–mesenchymal transition involved in the loss of the stem cell niche [8]. An upregulation of MHC class I and II molecules also accompanies the process [7,9]. Involvement of aberrant innate immune responses has been discussed, but the initial trigger remains unknown. Among possible aggravating external factors, studies have mostly focused on the use of cosmetics or sunscreen [9,10,11].

The possible impact of bacterial colonization has not received much attention so far. The idea that the hair follicle (HF) microbiome and interactions across the epithelium may contribute to disease onset and propagation is intriguing [12,13] because the bulge region is located right below the infundibulum, which hosts a wide spectrum of microbial communities [14,15]. The skin barrier in those deeper parts is considered more permeable than the fully developed stratum corneum near the skin surface. The practical relevance of the HF infundibulum as a reservoir, but also as a site for enhanced immune cell trafficking and antigen recognition, is well established [16,17,18]. Whether dysregulated cross-talk at this interface could contribute to inflammatory hair diseases remains to be determined [19]. For example, in the neutrophilic cicatricial alopecia subtype folliculitis decalvans (FD), *Staphylococcus aureus* colonization, along with a deficient host immune response, are recognized pathogenetic factors [20,21]. Beyond their typical role in infection or clinically relevant inflammation, evidence has been emerging that cutaneous microbes help calibrate the responses of innate and adaptive immune cells [22], and that HFs provide the environment for such calibration, affecting tissue homeostasis and HF cycling, e.g., via the involvement of regulatory T cells [22,23].

In this study, we investigate the presence of bacteria on the skin surface and in deeper parts of HFs from lesional and non-lesional scalps of LPP and FFA patients in comparison to healthy individuals, and patients with alopecia areata circumscripta (AAc). The study was complemented by immunohistochemical staining and ELISA analyses for human beta-defensins (HβD1, HβD2) and interleukin-17A (IL-17A).

## 2. Materials and Methods

This investigator-initiated exploratory study was conducted according to the guidelines of the Declaration of Helsinki and approved by the Charité-Universitätsmedizin Berlin Ethics Committee (protocol code EA1/113/18 and date of approval: 16 August 2020). Informed consent was obtained from all subjects involved in the study.

### 2.1. Study Population

Expanding our first exploratory metagenomic study on twelve healthy volunteers (six men, six women) with no scalp disease, aged 18–47 years [15] that act here as our control group, we also included twenty-one female alopecia patients (*n* = 6 FFA, *n* = 6 LPP, *n* = 7 AAc), aged 21–87 years. An established clinical diagnosis confirmed by a study physician using trichoscopy, and a positive pull-test [24] in the affected area were inclusion criteria for patients’ enrollment.

Recruitment criteria, scalp photography, skin physiological measurements, sampling procedures, samples processing, next-generation sequencing, ELISA quantification and the histology staining of scalp tissues were as described previously [15], except for a different metagenomics analysis.

Briefly, individuals with known hormonal imbalance, chronic viral infection, diabetes mellitus, or other concurrent inflammatory skin conditions were excluded. To assess the disease microbiome in an untreated state, subjects were excluded if they had received systemic retinoids, immunosuppressives, or immunomodulators within a month, antibiotics in the last 3 months, or topical treatment with corticosteroids within 3 weeks. Demographics (gender, age, height, weight, Fitzpatrick skin type) were documented, along with the dermatology life quality index (DLQI) [25] and disease severity scores [26,27,28,29].

The sampling and clinical procedures were performed on two scalp sites, i.e., frontal and occipital in healthy individuals, or lesional (hair-baring edges of active lesions) and non-lesional in patients.

During hair plucking and HF processing, we used designated sterile forceps/scalpels for each compartment to avoid cross-contamination; all instruments were disinfected with 70% ethanol [30], and surfaces, workstations, and equipment were decontaminated using 70% ethanol followed by DNA-ExitusPlus™ (AppliChem GmbH, Darmstadt, Germany). Reagent-only samples were added in every extraction as negative controls, to ensure that there was no cross-contamination during HFs-processing. Similarly, a moist swab exposed to room air for 20 s served as a negative control for the swab-processing.

### 2.2. Metagenomic Analysis

After demultiplexing, paired-end reads for the 227 human microbiome samples derived from patients (134 samples) and healthy individuals (93 samples) were imported into QIIME2.

The following steps were performed using the QIIME2 pipeline:

1. Sequence quality control and feature table construction: basic sequencing quality and further noise removal were achieved using the DADA2 QIIME2 plugins;
2. Tree generation for phylogenetic diversity analyses: a rooted phylogenetic tree was generated using the mafft program to perform a multiple sequence alignment. The alignment was filtered to remove highly variable positions. FastTree was then applied to generate a phylogenetic tree from the masked alignment. Finally, midpoint rooting was applied to place the root of the tree;
3. Taxonomic analysis: a pre-trained naive Bayes classifier was used to explore the taxonomic distribution of the samples. This classifier was trained on the SILVA (release v132, 2017) 99% 16S rRNA Operational Taxonomic Units (OTUs), on full length sequences of the 16S gene [31];
4. Alpha rarefaction plotting: alpha diversity was explored as a function of sampling depth using the QIIME2 diversity alpha-rarefaction visualizer;
5. Count table generation: finally, the results in BIOM format generated by QIIME2 were processed into count tables for further statistical analysis and processing.

The count tables were processed along with the corresponding taxonomy table and sample metadata using R v3.10. and phyloseq, microbiome, and microbiomeSeq packages [32,33,34,35] (Tables S1 and S2). Initially, OTUs classified to *Salinibacter ruber* (spike-in control culture) were identified and removed from the count table. Composition analysis was performed by aggregating OTU counts at the phylum and genus level, followed by transforming the counts to relative abundance. Composition plots were drawn after selecting the top five genera based on relative abundance, while aggregating the remaining taxa into “other”.

Differential abundance of genera between groups was calculated using the DeSeq2 package in R [34]. Although originally developed for probing differential expression in RNAseq, its negative binomial model was also applied for 16s rRNA [36]. Multiple testing adjustments were performed by the package’s false discovery rate (FDR) correction (Benjamini–Hochberg), while constraining the number of tests for the top five genera in overall abundance across all samples, by filtering out rare species with <500 counts across all samples. The pairwise tests were ran for healthy against the lesional and non-lesional samples from each disease entity.

### 2.3. Statistical Analysis

For the ELISA quantification data, IL-17A and HβD2 levels were analyzed using ANOVA with a least significant difference (LSD), using MATLAB 9.9 (R2020b) software with the Statistics and Machine Learning Toolbox (The MathWorks, Inc., Natick, MA, USA), for comparisons between groups. A *p*-value < 0.05 (*) was considered to be statistically significant.

### 2.4. Histological Assessment

Hematoxylin and eosin, Giemsa and immunohistochemical stainings were performed on 2–4 µm sections of formalin-fixed, paraffin-embedded diagnostic biopsies obtained from lesional scalp sites of 22 alopecia patients (*n* = 11 LPP, *n* = 8 FFA). Sections for Gram staining were cooked at 60 °C for 20 min, cooled at room temperature, deparaffinized, rehydrated, and stained following the manufacturer’s protocol (Sigma-Aldrich, Munich, Germany). Immunohistochemical staining for CD3, CD4, CD8, FOXP3, IL-17A, HβD1 and HβD2 were performed according to manufacturers’ recommendations, as described before [15]. Cell populations were counted using the Cell Counter Plugin in ImageJ. For negative controls, the primary antibody was omitted. When present, the infundibulum, central part (including bulge), suprabulbar (proximal), and bulb areas were analyzed in three fields (×400) per compartment, for up to three HFs per paraffin section. Staining for bacteria, HβD1 and HβD2 was assessed semi-quantitatively as grade 0 for no staining; grade 1 for weak staining; grade 2 for fair staining; and grade 4 for strong staining. The antibodies used, as well as the histological staining and assessment of the scalp specimens obtained from healthy volunteers i described in a prior publication [15]. 

## 3. Results

The demographic characteristics, skin physiological parameters (i.e., pH and sebum secretion), results from the DLQI, and disease severity scores are summarized in Table 1. Macrophotography and dermatoscopy, as shown in representative images in Figure 1, confirmed the clinical diagnoses and helped identify active lesions.

Mean age, mean weight, mean height, Fitzpatrick skin type, known atopic dermatitis or psoriasis, scalp pH, and sebum measurements from two scalp areas. DLQI scores: 0–1, no effect at all; 2–5, small effect; 6–10, moderate effect; 11–20, very large effect; 21–30, extremely large effect on patient’s life. ^1^Disease severity scores used for each disease entity: Severity of Alopecia Tool (SALT) Score for AA; Lichen Planopilaris Activity Index (LPPAI) Score for LPP; Frontal Fibrosing Alopecia Severity Index (FFASI) Score for FFA. AAc, alopecia areata circumscripta; LPP, lichen planopilaris; FFA, frontal fibrosing alopecia; SD, standard deviation; DLQI, Dermatology Life Quality Index.

**Figure 2:**
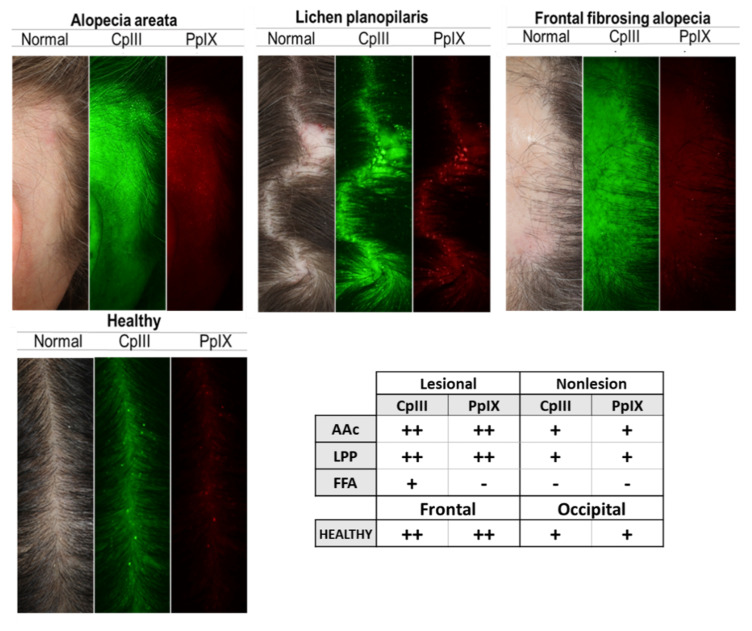
*Cutibacterium acnes* presence on the scalp surface of healthy subjects and alopecia patients. Porphyrins, *Cutibacterium acnes* excretions, fluoresce in UV light, and exhibit circular white spot characteristics. Images from left to right: Standard White Light Image (Clinical Photograph) of the scalp surface (follicular opening); Coproporphyrin (CpIII) Fluorescence Image; and Protoporphyrin (PpIX) Fluorescence Image. Green fluorescence spots correspond to CpIII; red fluorescence spots correspond to PpIX. Table shows the results of the analysis of CpIII and PpIX presence on the scalp, using VISIA CR images. Porphyrins are less numerous on FFA patients. Fluorescence dots × field of view: +, 50–150; ++, 150–250; +++, >250. AAc (*n* = 7), LPP (*n* = 6), FFA (*n* = 6), Healthy (*n* = 12). LPP, lichen planopilaris; FFA, frontal fibrosing alopecia; AAc, alopecia areata circumscripta.

VISIA imaging, typically used to visualize porphyrins as indicators of metabolic activity of *Cutibacterium acnes* in facial skin, revealed prominent fluorescent spots in the frontal area of the healthy scalp and in lesional sites of patients’ scalp, except for FFA (Figure 2). Concerning the relative abundance of bacteria, the metagenomic analyses of swabs and plucked HFs identified Firmicutes, Actinobacteria, and a smaller percentage of Proteobacteria, as the three main phyla in healthy individuals and patients. The mean count of reads per sample was highest for samples obtained from the middle compartment of plucked HFs (~2 M), an order of magnitude lower for swabs (~200 K) and even lower for HF bulbs (~20 K). 

**Figure 3:**
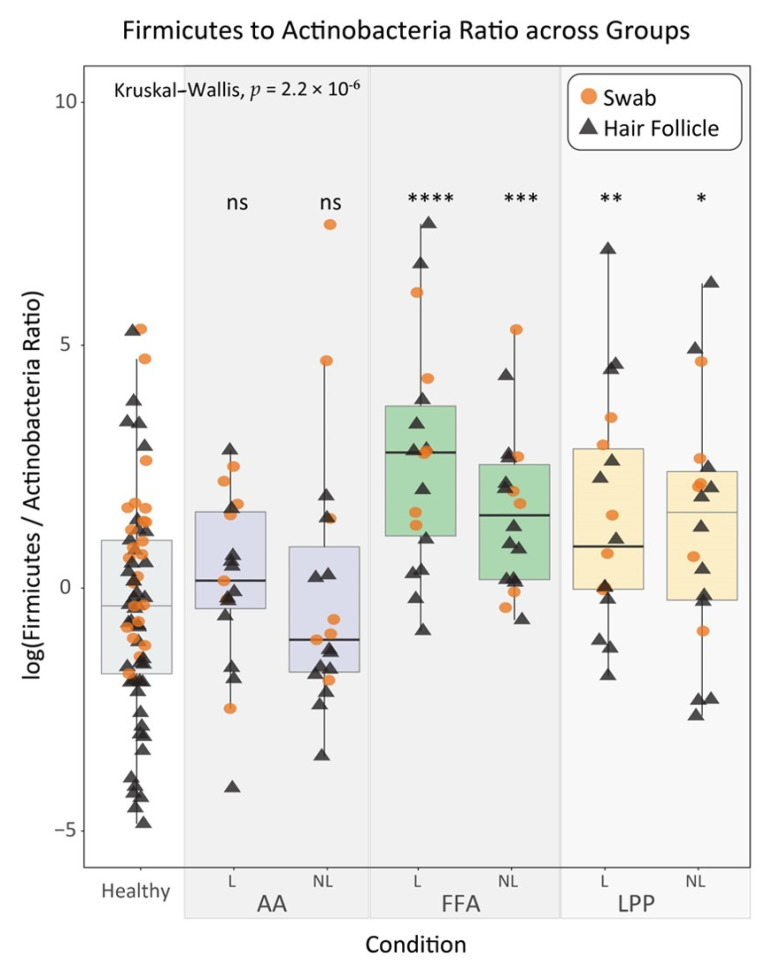
The statistical analysis suggests a trade-off between the phyla Firmicutes and Actinobacteria and the diseases under study. log(FAR) pairwise comparisons between healthy and all disease subgroups (L for lesional and NL for non-lesional scalp sites). FAR comparisons between healthy and lesional sites show a notable increase in all diseases. In cicatricial alopecia, this difference is statistically significant (*p*-value < 0.05). Non-lesional FFA and LPP sites also exhibit significantly increased values. Orange circles and black triangles represent swab and scalp HF samples, respectively. ns indicates *p*-value > 0.05, * < 0.05, ** < 0.01, *** < 0.001 and **** < 0.0001 from Mann–Whitney U test comparing each subgroup with the reference healthy group. FAR, actinobacteria and firmicutes ratio; AA, alopecia areata circumscripta; FFA, frontal fibrosing alopecia; LPP, lichen planopilaris; ns, not significant.

At the phylum level, Firmicutes and Actinobacteria were equally present in the scalp swabs from healthy and non-lesional sites of AAc and FFA patients, whereas Firmicutes dominated non-lesional LPP swabs and all lesional alopecia swabs (>70%). Deeper along the HFs, these abundances changed. Actinobacteria dominated the healthy HFs and non-lesional HFs from AAc, but markedly decreased in lesional AAc (~50%), non-lesional FFA (~25%) and LPP (~7.5%), and lesional FFA and LPP (>5%) plucked HFs. This steep decline was accompanied by a sharp increase in the Firmicutes relative abundance. The difference in the Firmicutes to Actinobacteria ratio compared to healthy reached statistical significance in all lesional sites of cicatricial alopecia and non-lesional FFA and LPP sites (*p*-value < 0.05) (Figure 3). 

More detailed analyses were performed at genus resolution. No major gender-related or regional difference in the microbial composition of healthy samples were found in any of the downstream analyses. Likewise, comparing the α-diversities across the groups did not reveal any statistically significant differences (Figure S1).

**Figure 4:**
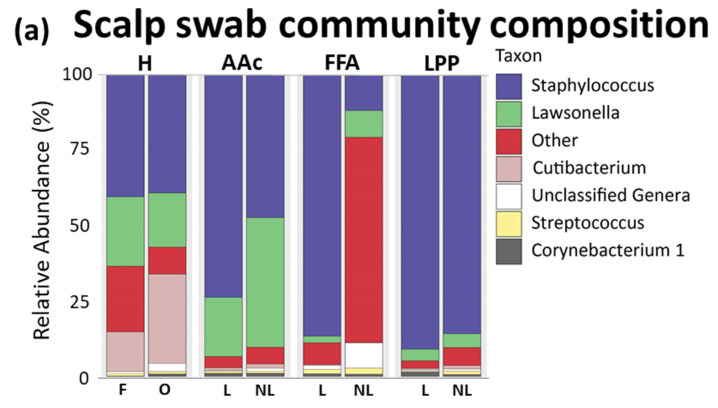

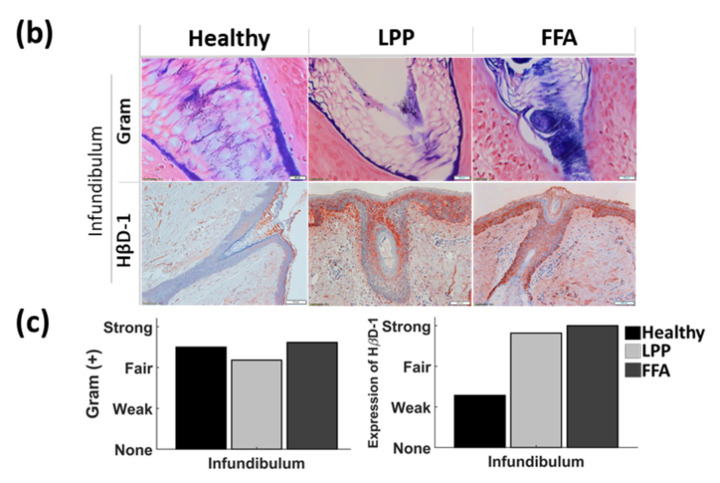
Bacterial profiling of the scalp surface of healthy subjects and alopecia patients. (**a**) Community composition of the scalp surface of healthy subjects and alopecia patients at the Genus level. Relative abundance (%) of the most abundant bacteria collected from scalp swabs, sampled from frontal (F) and occipital (O) scalps in healthy or lesional (L) and non-lesional (NL) scalp regions in patients. H (*n* = 12), AAc (*n* = 6), LPP (*n* = 6), FFA (*n* = 6). (**b**) Representative images at the level of HF infundibulum. Gram staining and HβD1 expression in paraffin-embedded sections of healthy scalp, and lesional scalp of cicatricial alopecia patients, assessed by immunohistochemistry. Magnification for Gram images × 400, bar scale = 20 μm, and for HβD1 images × 100, bar scale = 100 μm, respectively. (**c**) Semi-quantitative analysis of bacteria presence and the expression of HβD1 antimicrobial peptide at the level of infundibulum verifies the strong microbial colonization in HF openings and scalp surface. H (*n* = 3), LPP (*n* = 8), FFA (*n* = 7). H, healthy; FFA, frontal fibrosing alopecia; LPP, lichen planopilaris; AAc, alopecia areata circumscripta; HF, hair follicle.

**Figure 5:**
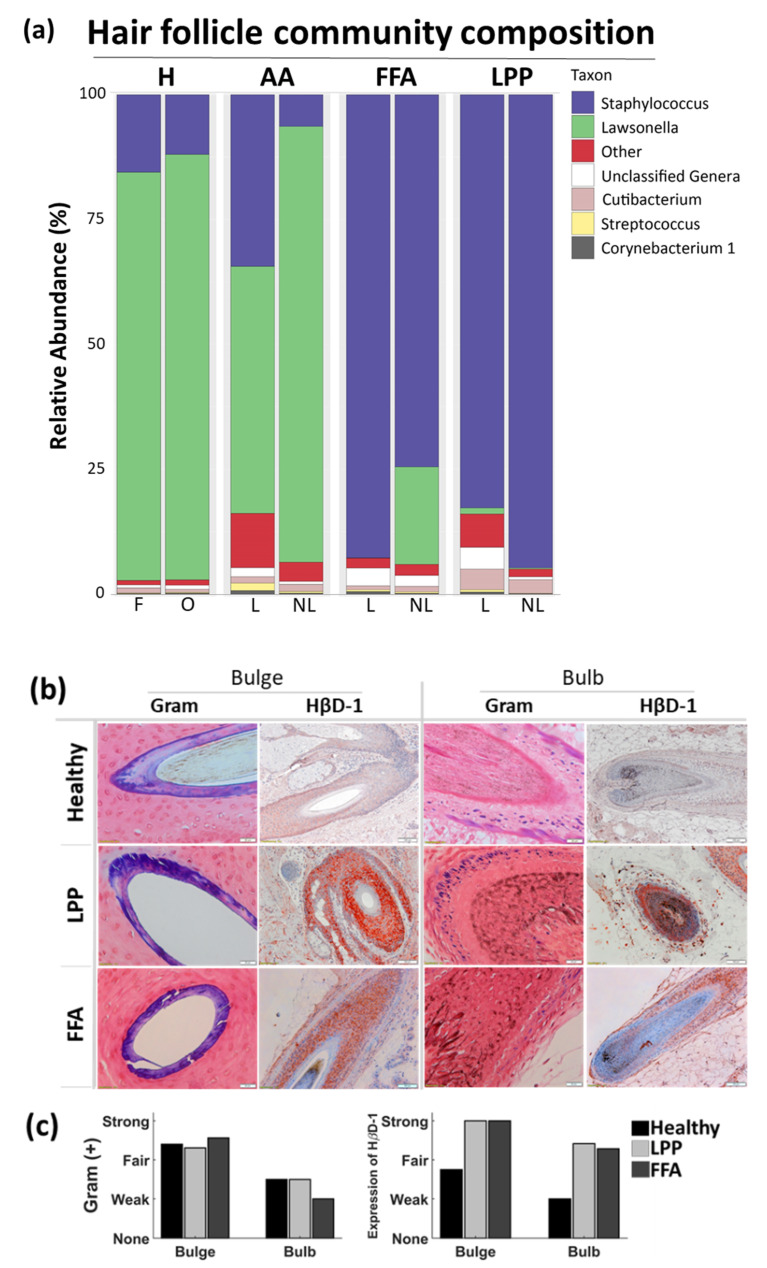
Bacterial presence below the scalp surface, along the middle and lower hair follicle of healthy subjects and alopecia patients. (**a**) HF community composition at the genus level. Relative abundance (%) of the most abundant bacteria in hair follicles, plucked from frontal (F) and occipital (O) scalp in healthy and lesional (L) and non-lesional (NL) scalp regions in patients. H (*n* = 12), AAc (*n* = 6), LPP (*n* = 6), FFA (*n* = 6). (**b**) Representative histology images of the middle HF, at the level of the bulge region and bulb. Gram staining and HβD1 expression in paraffin-embedded sections of healthy scalp and lesional scalp of cicatricial alopecia patients, assessed by immunohistochemistry. Magnification for Gram images × 400, bar scale = 20 μm, and for HβD1 images × 100, bar scale = 100 μm, respectively. (**c**) Semi-quantitative analysis of the bacteria presence and the expression of HβD1 antimicrobial peptide at the bulge level and bulbs verifies that microbial colonization extends to lower follicular compartments below the scalp surface, following a progressive decrease towards the bulbs. H (*n* = 3), LPP (*n* = 7), FFA (*n* = 8). H, healthy; FFA, frontal fibrosing alopecia; HF, hair follicle; LPP, lichen planopilaris; AAc, alopecia areata circumscripta.

**Figure 6:**
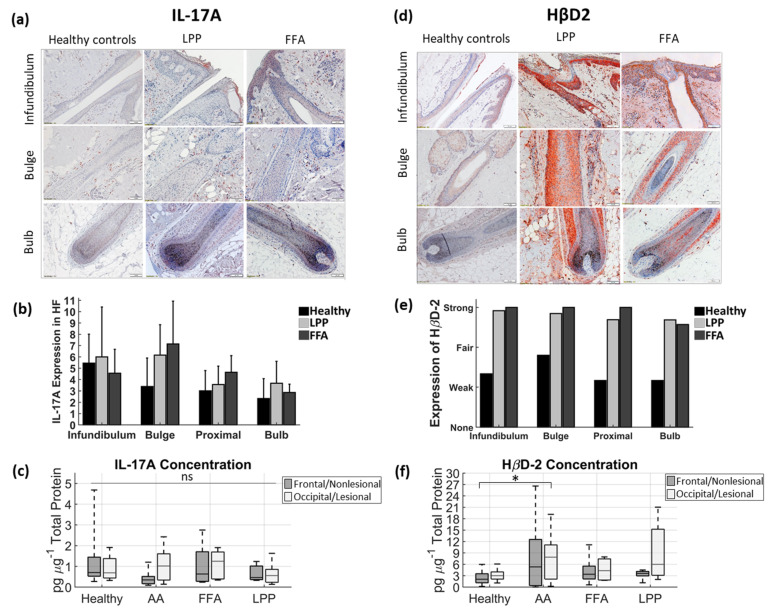
The expression of IL-17A and HβD2 in scalp lesions of patients with cicatricial alopecia and healthy subjects: (**a**,**b**) IL-17A; and (**d**,**e**) HβD-2 expression in paraffin-embedded sections of healthy scalp and cutaneous lesions (scalp) of cicatricial alopecia patients assessed by immunohistochemistry, followed by a semi-quantitative analysis. The figure panel pair images taken at different hair follicle depths of interest: infundibulum, central part (including bulge region) and bulb. Magnification × 100, scale bars = 100 μm. For (**b**) IL-17A, the *y*-axis shows the mean number of positive cells per follicular compartment. Marked presence of (**c**) IL-17A and (**f**) HβD2 in infra-infundibular compartments was further confirmed by ELISA analyses of plucked hair follicles from frontal and occipital scalps in healthy and lesional and non-lesional scalp areas in patients with AA (*n* = 7), LPP (*n* = 6) and FFA (*n* = 6). In each box of the box plot, the central line indicates the median, and the bottom and top edges of the box indicate the 25th and 75th percentiles, respectively. The whiskers extend to the maximum and minimum data values. LPP; lichen planopilaris, AA; alopecia areata circumscripta, FFA; frontal fibrosing alopecia; ns, not significant; * *p* < 0.05 AA (lesional site) compared to healthy controls (frontal site).

The genera distribution in swab samples and samples of bacterial DNA extracted from plucked HFs, are displayed separately, each in relation to HβD1 immunohistochemistry and Gram staining (Figure 4 and Figure 5), while HβD2 and IL-17A expression are displayed in Figure 6, along with ELISA results from protein extracts.

### 3.1. Scalp Surface

In swabs from healthy individuals, the three dominating genera were Staphylococcus (~40%), followed by Lawsonella (~20%), and Cutibacterium (13% frontal, 29% occipital region). In lesional sites of all alopecia patients, Staphylococcus exceeded other genera (>70%). In swabs from non-lesional sites, Staphylococcus was also dominant in LPP patients (85%), whereas abundances varied in AAc (45%), and FFA (11%) patients. When comparing lesional and non-lesional relative abundances of Lawsonella, we found notable differences in AAc (44% vs. 20%) and FFA (10% vs. 3%) (Figure 4a, Table S3). 

The positive Gram staining and the HβD expression in the infundibulum are in line with the microbial presence in the HF openings, particularly in patients (Figure 4b,c and Figure 6d,e).

### 3.2. Below the Surface

In plucked HF samples, the two most represented genera were Lawsonella and Staphylococcus. Lawsonella dominated healthy and non-lesional AAc samples with a relative abundance of >80%, but also lesional AAc HFs (57%). Staphylococcus dominated lesional and non-lesional hair samples of FFA and LPP patients (>75%), but only to a lesser extent in lesional AAc samples (35%). The differential abundance analysis confirmed statistically significant shifts for Staphylococcus and Lawsonella in nearly all pairwise comparisons between hair samples from healthy individuals and lesional alopecia sites (Table S4).

Positive Gram staining was observed in compartments near the bulge, but progressively decreased in lower regions (bulb) (Figure 5b,c). The strong staining for HβD all along the outer root sheath of the HF, even in bulbar regions, was much more pronounced in samples from cicatricial alopecia patients (Figure 5b,c and Figure 6d,e). When quantified by ELISA, we observed increased IL-17A in lesional AAc sites, compared to non-lesional, while lesional LPP HF samples contained more HβD2 than non-lesional sites (Figure 6c,f).

**Figure 7:**
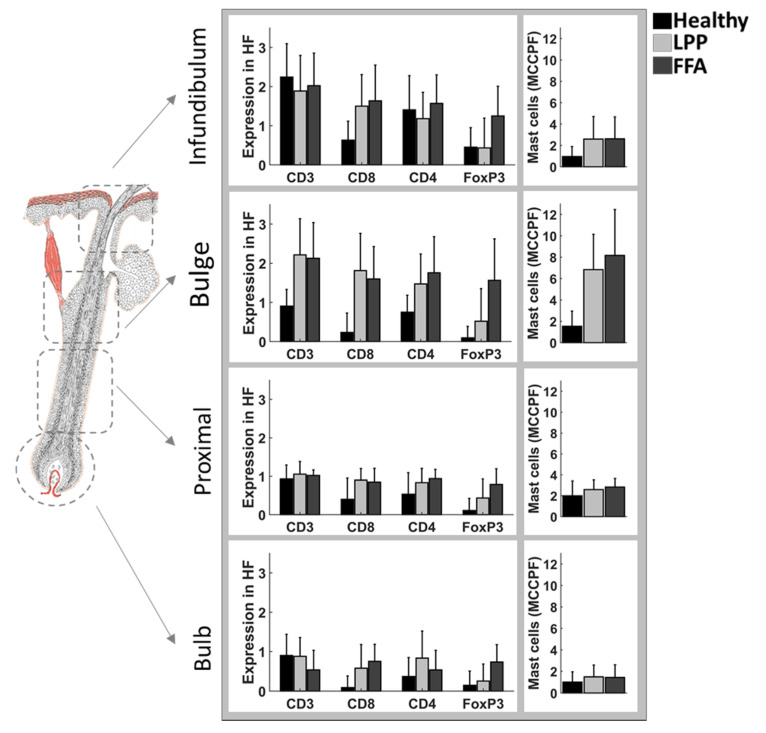
Semiquantitative analysis of CD3+, CD4+, CD8+, FoxP3+ cells and mast cells. Semi-quantitative analysis of antibodies against CD3+, CD4+, CD8+, FoxP3+ cells and mast cells (Giemsa staining), in the different follicular compartments of interest and their corresponding perifollicular mesenchymal compartments, representing the connective tissue sheath around each follicular compartment; infundibulum, bulge region, proximal area, and bulb. For mast cells, the *y*-axis shows the mean number of mast cells per follicular and perifollicular compartment. For CD3+, CD4+, CD8+ and FoxP3+ expression was scored as follows: 0, no expression; 1, mean cell count per field (MCCPF) between 1 and 10; 2, MCCPF between 10 and 20; or 3, MCCPF more than 20. H (*n* = 3), LPP (*n* = 11), FFA (*n* = 8). LPP, lichen planopilaris; FFA, frontal fibrosing alopecia.

On the cellular level, CD3+, CD4+, CD8+, FOXP3+ and mast cell staining confirmed the infundibulo–isthmic area, including the bulge, as the main site of inflammation, with a shift towards CD8+ T cells in LPP and FFA (Figure 7).

## 4. Discussion

Our results give insights into the relative abundances of bacteria and the presence of HβD and IL-17A in patients with FFA and LPP, compared to healthy controls and AAc patients. In line with previous studies, the phyla Actinobacteria, Firmicutes and Proteobacteria, and the genera Staphylococcus, Lawsonella, and Cutibacterium were the dominating taxa found on healthy skin [37]. Our analyses on the phylum and genus level are consistent; for example, the dominance of Firmicutes in cicatricial alopecia agrees with the Staphylococcus dominance at the genus level, where Actinobacteria and Lawsonella are under-represented. The conflicting results of our VISIA analysis and the next-generation sequencing results are not surprising, because the two are not comparable to start with. VISIA imaging is quantitative because the fluorescent spots directly represent bacterial material, whereas our next-generation sequencing compares the relative abundances of bacteria within and across samples, meaning that the presented metagenomic results do not represent the actual number of bacteria present within the samples.

Over-representation of Firmicutes is widely observed in inflammatory skin diseases including psoriatic lesions and neutrophilic disorders such as hidradenitis suppurativa [38,39,40,41]. Similarly, in previous reports, most bacterial sequences obtained from samples of FD patients belonged to Staphylococcus, which corresponds to our current understanding of the disease-aggravating role of *Staphylococcus aureus* in FD [20,41,42]. In atopic dermatitis, *Staphylococcus aureus* is critically involved in immunological signaling across the skin barrier [43]. Although there are first reports of microbial shifts in AAc patients [44], we found areata samples considerably more similar to healthy controls than scarring alopecia samples. Interestingly, in LPP and FFA, the Staphylococcus dominance extended to deeper compartments, whereas in healthy and AAc samples, Lawsonella became more prevalent.

The Firmicutes to Actinobacteria ratio could be a measure for dysbiosis in FFA and LPP; it revealed a strong positive correlation compared to healthy individuals. While such a ratio is not yet established for the skin microbiome, a similar concept using the Firmicutes/Bacteroidetes ratio is a commonly used marker of dysbiosis in inflammatory bowel diseases [45]. Surprisingly, no significant deviations were observed in the diversity measures between healthy and diseased states. This may be due to the limited number of species affected or the relatively limited sample size.

It remains unclear whether microbiota shifts have any connection, causative or epiphenomenal, to the localization of the inflammatory infiltrate along the HF. Interestingly, however, we found positive staining for HβD2, in infrainfundibular and bulb areas of HFs. In the interfollicular epidermis, effective triggering in response to pathogen contact was shown for Gram-negative (*Pseudomonas aeruginosa*, *Escherichia coli*) and Gram-positive (*S. aureus*) bacteria and yeast (*Candida albicans*) [46,47]. HβD1 and ΗβD2 are known to attract T cells and immature dendritic cells, while ΗβD2 induces mast cell migration [48]. On the other hand, lichenoid inflammation itself can be a strong driver of HβD expression [49,50]. While the molecular interactions within the HF are poorly characterized, a complex role of beta-defensins has been established for gingiva and periodontal pockets, sites of ongoing microbe exposure, which are also frequently affected by lichenoid inflammation [51]. At these exposed sites, beta-defensins were shown to regulate host reactions, but also act as immunoregulatory mediators helping to contain inflammation [52].

Our study had two inherent limitations. Firstly, all the patients were female, whereas the control group consisted of both male and female subjects. However, some preliminary analyses of our data excluding the male participants only slightly changed the bacterial relative abundances of our healthy control group (Figure S2). Therefore, for the full metagenomic analysis, we decided to keep our healthy population as it was.

Moreover, there was a mean age difference between the patient and healthy groups. Changes in the composition of the skin residential bacteria with ageing have been proposed; therefore, one can assume that this age gap could explain the bacterial shifts described herein. Even though these bacterial changes of the scalp have not been clearly defined in the literature yet, age-related changes in the skin microbiota have been observed [53,54,55,56]. For example, while Firmicutes and Proteobacteria were more abundant on the forehead of Japanese women in the older group compared to the younger group, Actinobacteria was the most abundant [53]. On the forehead of western European women and the scalp of Japanese women, the Firmicutes abundance was similar amongst the two groups, whereas Proteobacteria was increased in older women [53,54]. On the contrary, the bacterial analysis of the forehead of Korean women and the cheeks of Chinese women revealed that Firmicutes was more abundant in the younger group [55,56]. However, our older group of patients did not share similar findings. Therefore, based on the current literature, our finding that there is a shift in bacterial colonization in FFA and LPP patients towards a Staphylococcus dominance cannot be explained by the age difference between the groups.

Although IL-17 has been discussed as a mediator in lichenoid inflammation [57,58], neither immunohistochemistry nor protein extracts support a dominance of this cytokine in our LPP samples, while the enhanced IL-17 found in plucked HFs from active lesional AAc sites is in accordance with the recognized dominant role of TH17 immune responses in AAc [59]. However, the high presence of HβD2 in combination with less pronounced IL-17 production may be connected, because HβD2 was shown to downregulate IL-17 expression in T cells [60].

It remains unclear whether the initiating events for the inflammatory cascades involved in LPP and FFA are driven from within the HF epithelium, or to what extent this crosstalk with bacteria on the outside of the epithelium plays a role. However, the approach presented herein provided consistent results and represents a starting point for further analyses. Investigating how microbial diversities impact HF cycling and regeneration, and their pathogenetic relevance in inflammatory scalp diseases, can ultimately have practical implications in finding novel management strategies and therapeutic targets to reshape the microbiome towards a healthy status.

## Figures and Tables

**Table 1:** Subject demographics and clinical data collected.

Number of Subjects	Healthy Controls		AAc		LPP		FFA	
	*n* = 12	%	*n* = 7	%	*n* = 6	%	*n* = 6	%
**Male**	6	50	0	0	0	0	0	0
**Female**	6	50	7	100	6	100	6	100
**Mean age, years (SD)**	28.7 (7.2)		41.3 (15.9)		54.8 (18.1)		56.8 (7.4)	
**Mean Weight, kg (SD)**	67.1 (10.8)		70 (6.5)		62.7 (17.8)		71.8 (7.7)	
**Mean Height, cm (SD)**	170.8 (10.2)		171.4 (4.2)		166 (6.4)		167.4 (7.1)	
**Atopic Dermatitis**	0		0		0		0	
**Psoriasis**	0		0		0		0	
**Fitzpatrick Phototype**								
**Type II**	5	42%	2	29%	1	17%	1	17
**Type II/III**	0	0%	0	0%	1	17%	1	17
**Type III**	3	25%	3	43%	4	67%	1	33
**Type III/IV**	0	0%	2	29%	0	0%	0	0
**Type IV**	3	25%	0	0%	0	0%	2	33
**Type V**	1	8%	0	0%	0	0%	0	0
**Scalp pH (SD)**								
**Frontal**	6 (1.5)		n/a		n/a		n/a	
**Occipital**	5.7 (1.2)		n/a		n/a		n/a	
**Affected**	n/a		6.9 (1.1)		6.8 (0.8)		6.5 (0.8)	
**Not Affected**	n/a		6.9 (2)		6.9 (1.3)		6.3 (0.6)	
**Scalp Sebum (SD)**								
**Frontal**	107.4 (96.5)		n/a		n/a		n/a	
**Occipital**	78.8 (73.6)		n/a		n/a		n/a	
**Affected**	n/a		44.3 (49.5)		71.3 (65.9)		29 (39.1)	
**Not Affected**	n/a		16.1 (14.5)		60.2 (20.1)		41 (37.9)	
**DLQI (mean, SD)**	n/a		5 (3.8)		5.8 (2.1)		4.2 (3.7)	
**Severity Score^1^ (mean, SD)**	n/a		20.3 (15.2)		6.6 (2.4)		33.9 (6.2)	

## Data Availability

The datasets generated during and/or analyzed during the current study are available from the corresponding author on reasonable request.

## Supplementary Materials

The following are available online at https://www.mdpi.com/2227-9059/9/3/266/s1, Figure S1. Alpha diversity comparison plot; Figure S2. Preliminary data analyses excluding the male population of our control group; Table S1. Count tablel Table S2. Taxonomy table_Silva; Table S3. Top 5 in total counts_Swabs; Table S4. Top 5 in total counts_HF.

## References

[1] Vano-Galvan S., Saceda-Corralo D., Blume-Peytavi U., Cucchia J., Dlova N.C., Gavazzoni Dias M.F.R., Grimalt R., Guzman-Sanchez D., Harries M., Ho A. (2019). Frequency of the Types of Alopecia at Twenty-Two Specialist Hair Clinics: A Multicenter Study. Skin Appendage Disord.

[2] MacDonald A., Clark C., Holmes S. (2012). Frontal fibrosing alopecia: A review of 60 cases. J. Am. Acad. Dermatol..

[3] Kanti V., Constantinou A., Reygagne P., Vogt A., Kottner J., Blume-Peytavi U. (2019). Frontal fibrosing alopecia: Demographic and clinical characteristics of 490 cases. J. Eur. Acad. Dermatol. Venereol..

[4] Saceda-Corralo D., Pindado-Ortega C., Moreno-Arrones O.M., Ortega-Quijano D., Fernandez-Nieto D., Jimenez-Cauhe J., Vano-Galvan S. (2020). Association of Inflammation With Progression of Hair Loss in Women With Frontal Fibrosing Alopecia. JAMA Dermatol..

[5] Cerqueira E.R., Valente N., Sotto M.N., Romiti R. (2016). Comparative Analysis of Immunopathological Features of Lichen Planopilaris and Female Patients with Frontal Fibrosing Alopecia. Int. J. Trichology.

[6] Galvez-Canseco A., Sperling L. (2018). Lichen planopilaris and frontal fibrosing alopecia cannot be differentiated by histopathology. J. Cutan. Pathol..

[7] Chen C.L., Huang W.Y., Wang E.H.C., Tai K.Y., Lin S.J. (2020). Functional complexity of hair follicle stem cell niche and therapeutic targeting of niche dysfunction for hair regeneration. J. Biomed. Sci..

[8] Imanishi H., Ansell D.M., Cheret J., Harries M., Bertolini M., Sepp N., Biro T., Poblet E., Jimenez F., Hardman J. (2018). Epithelial-to-Mesenchymal Stem Cell Transition in a Human Organ: Lessons from Lichen Planopilaris. J. Invest. Dermatol..

[9] Harries M.J., Jimenez F., Izeta A., Hardman J., Panicker S.P., Poblet E., Paus R. (2018). Lichen Planopilaris and Frontal Fibrosing Alopecia as Model Epithelial Stem Cell Diseases. Trends Mol. Med..

[10] Aldoori N., Dobson K., Holden C.R., McDonagh A.J., Harries M., Messenger A.G. (2016). Frontal fibrosing alopecia: Possible association with leave-on facial skin care products and sunscreens; a questionnaire study. Br. J. Dermatol..

[11] Tavakolpour S., Mahmoudi H., Abedini R., Kamyab Hesari K., Kiani A., Daneshpazhooh M. (2019). Frontal fibrosing alopecia: An update on the hypothesis of pathogenesis and treatment. Int. J. Womens Dermatol..

[12] Polak-Witka K., Rudnicka L., Blume-Peytavi U., Vogt A. (2019). The role of the microbiome in scalp hair follicle biology and disease. Exp. Dermatol..

[13] Constantinou A., Kanti V., Polak-Witka K., Blume-Peytavi U., Spyrou G.M., Vogt A. (2021). The Potential Relevance of the Microbiome to Hair Physiology and Regeneration: The Emerging Role of Metagenomics. Biomedicines.

[14] Nakatsuji T., Chiang H.I., Jiang S.B., Nagarajan H., Zengler K., Gallo R.L. (2013). The microbiome extends to subepidermal compartments of normal skin. Nat. Commun..

[15] Polak-Witka K., Constantinou A., Schwarzer R., Helmuth J., Wiessner A., Hadam S., Kanti V., Rancan F., Andruck A., Richter C. (2020). Identification of anti-microbial peptides and traces of microbial DNA in infrainfundibular compartments of human scalp terminal hair follicles. Eur. J. Dermatol..

[16] Lademann J., Knorr F., Richter H., Blume-Peytavi U., Vogt A., Antoniou C., Sterry W., Patzelt A. (2008). Hair follicles--an efficient storage and penetration pathway for topically applied substances. Summary of recent results obtained at the Center of Experimental and Applied Cutaneous Physiology, Charite -Universitatsmedizin Berlin, Germany. Skin Pharmacol. Physiol.

[17] Blume-Peytavi U., Vogt A. (2011). Human hair follicle: Reservoir function and selective targeting. Br. J. Dermatol..

[18] Chen C.C., Plikus M.V., Tang P.C., Widelitz R.B., Chuong C.M. (2016). The Modulatable Stem Cell Niche: Tissue Interactions during Hair and Feather Follicle Regeneration. J. Mol. Biol..

[19] Vogt A., Constantinou A., Rancan F., Ghoreschi K., Blume-Peytavi U., Combadiere B. (2020). A niche in the spotlight: Could external factors critically disturb hair follicle homeostasis and contribute to inflammatory hair follicle diseases?. Exp. Dermatol..

[20] Otberg N., Kang H., Alzolibani A.A., Shapiro J. (2008). Folliculitis decalvans. Dermatol. Ther..

[21] Chiarini C., Torchia D., Bianchi B., Volpi W., Caproni M., Fabbri P. (2008). Immunopathogenesis of folliculitis decalvans: Clues in early lesions. Am. J. Clin. Pathol..

[22] Campbell D.J., Koch M.A. (2017). Living in Peace: Host-Microbiota Mutualism in the Skin. Cell Host Microbe.

[23] Scharschmidt T.C., Vasquez K.S., Pauli M.L., Leitner E.G., Chu K., Truong H.A., Lowe M.M., Sanchez Rodriguez R., Ali N., Laszik Z.G. (2017). Commensal Microbes and Hair Follicle Morphogenesis Coordinately Drive Treg Migration into Neonatal Skin. Cell Host Microbe.

[24] Hillmann K., Blume-Peytavi U. (2009). Diagnosis of hair disorders. Semin. Cutan. Med. Surg.

[25] Finlay A.Y., Khan G.K. (1994). Dermatology Life Quality Index (DLQI)—A simple practical measure for routine clinical use. Clin. Exp. Dermatol..

[26] Vano-Galvan S., Molina-Ruiz A.M., Fernandez-Crehuet P., Rodrigues-Barata A.R., Arias-Santiago S., Serrano-Falcon C., Martorell-Calatayud A., Barco D., Perez B., Serrano S. (2015). Folliculitis decalvans: A multicentre review of 82 patients. J. Eur. Acad. Dermatol. Venereol..

[27] Olsen E.A., Hordinsky M.K., Price V.H., Roberts J.L., Shapiro J., Canfield D., Duvic M., King L.E., McMichael A.J., Randall V.A. (2004). Alopecia areata investigational assessment guidelines--Part II. National Alopecia Areata Foundation. J. Am. Acad. Dermatol..

[28] Chiang C., Sah D., Cho B.K., Ochoa B.E., Price V.H. (2010). Hydroxychloroquine and lichen planopilaris: Efficacy and introduction of Lichen Planopilaris Activity Index scoring system. J. Am. Acad. Dermatol..

[29] Holmes S., Ryan T., Young D., Harries M., British H., Nail S. (2016). Frontal Fibrosing Alopecia Severity Index (FFASI): A validated scoring system for assessing frontal fibrosing alopecia. Br. J. Dermatol..

[30] Graziano M.U., Graziano K.U., Pinto F.M., Bruna C.Q., de Souza R.Q., Lascala C.A. (2013). Effectiveness of disinfection with alcohol 70% (w/v) of contaminated surfaces not previously cleaned. Rev. Lat Am. Enferm..

[31] Quast C., Pruesse E., Yilmaz P., Gerken J., Schweer T., Yarza P., Peplies J., Glockner F.O. (2013). The SILVA ribosomal RNA gene database project: Improved data processing and web-based tools. Nucleic Acids Res..

[32] Team, R.C. R: A Language and Environment for Statistical Computing. R Foundation for Statistical Computing. https://www.R-project.org.

[33] McMurdie P.J., Holmes S. (2013). phyloseq: An R package for reproducible interactive analysis and graphics of microbiome census data. PLoS ONE.

[34] Leo L., Shetty S.S. Tools for Microbiome Analysis in R. Microbiome Package Version. *Bioconductor* 2017–2019. http://microbiome.github.io/microbiome.

[35] Ssekagiri A., Sloan W.T., Ijaz U.Z. microbiomeSeq: An R package for analysis of microbial communities in an environmental context. Proceedings of the ISCB Africa ASBCB Conference.

[36] Ocejo M., Oporto B., Hurtado A. (2019). 16S rRNA amplicon sequencing characterization of caecal microbiome composition of broilers and free-range slow-growing chickens throughout their productive lifespan. Sci. Rep..

[37] Grice E.A., Kong H.H., Conlan S., Deming C.B., Davis J., Young A.C., Program N.C.S., Bouffard G.G., Blakesley R.W., Murray P.R. (2009). Topographical and temporal diversity of the human skin microbiome. Science.

[38] Fahlen A., Engstrand L., Baker B.S., Powles A., Fry L. (2012). Comparison of bacterial microbiota in skin biopsies from normal and psoriatic skin. Arch. Dermatol. Res..

[39] Haskin A., Fischer A.H., Okoye G.A. (2016). Prevalence of Firmicutes in Lesions of Hidradenitis Suppurativa in Obese Patients. JAMA Dermatol..

[40] Ring H.C., Riis Mikkelsen P., Miller I.M., Jenssen H., Fuursted K., Saunte D.M., Jemec G.B. (2015). The bacteriology of hidradenitis suppurativa: A systematic review. Exp. Dermatol..

[41] Sillani C., Bin Z., Ying Z., Zeming C., Jian Y., Xingqi Z. (2010). Effective treatment of folliculitis decalvans using selected antimicrobial agents. Int. J. Trichology.

[42] Annessi G. (1998). Tufted folliculitis of the scalp: A distinctive clinicohistological variant of folliculitis decalvans. Br. J. Dermatol..

[43] Leyden J.J., Marples R.R., Kligman A.M. (1974). Staphylococcus aureus in the lesions of atopic dermatitis. Br. J. Dermatol..

[44] Pinto D., Sorbellini E., Marzani B., Rucco M., Giuliani G., Rinaldi F. (2019). Scalp bacterial shift in Alopecia areata. PLoS ONE.

[45] Rinninella E., Raoul P., Cintoni M., Franceschi F., Miggiano G.A.D., Gasbarrini A., Mele M.C. (2019). What is the Healthy Gut Microbiota Composition? A Changing Ecosystem across Age, Environment, Diet, and Diseases. Microorganisms.

[46] Yamasaki K., Gallo R.L. (2008). Antimicrobial peptides in human skin disease. Eur. J. Dermatol..

[47] Ali R.S., Falconer A., Ikram M., Bissett C.E., Cerio R., Quinn A.G. (2001). Expression of the peptide antibiotics human beta defensin-1 and human beta defensin-2 in normal human skin. J. Invest. Dermatol..

[48] Niyonsaba F., Ogawa H., Nagaoka I. (2004). Human beta-defensin-2 functions as a chemotactic agent for tumour necrosis factor-alpha-treated human neutrophils. Immunology.

[49] Abiko Y., Jinbu Y., Noguchi T., Nishimura M., Kusano K., Amaratunga P., Shibata T., Kaku T. (2002). Upregulation of human beta-defensin 2 peptide expression in oral lichen planus, leukoplakia and candidiasis. An immunohistochemical study. Pathol. Res. Pract..

[50] Nishimura M., Abiko Y., Kusano K., Yamazaki M., Saitoh M., Mizoguchi I., Jinbu Y., Noguchi T., Kaku T. (2003). Localization of human beta-defensin 3 mRNA in normal oral epithelium, leukoplakia, and lichen planus: An in situ hybridization study. Med. Electron Microsc..

[51] Chung W.O., Dommisch H., Yin L., Dale B.A. (2007). Expression of defensins in gingiva and their role in periodontal health and disease. Curr. Pharm. Des..

[52] Gursoy U.K., Kononen E. (2012). Understanding the roles of gingival beta-defensins. J. Oral Microbiol..

[53] Shibagaki N., Suda W., Clavaud C., Bastien P., Takayasu L., Iioka E., Kurokawa R., Yamashita N., Hattori Y., Shindo C. (2017). Aging-related changes in the diversity of women’s skin microbiomes associated with oral bacteria. Sci. Rep..

[54] Juge R., Rouaud-Tinguely P., Breugnot J., Servaes K., Grimaldi C., Roth M.P., Coppin H., Closs B. (2018). Shift in skin microbiota of Western European women across aging. J. Appl. Microbiol..

[55] Kim H.J., Kim J.J., Myeong N.R., Kim T., Kim D., An S., Kim H., Park T., Jang S.I., Yeon J.H. (2019). Segregation of age-related skin microbiome characteristics by functionality. Sci. Rep..

[56] Kim M., Park T., Yun J.I., Lim H.W., Han N.R., Lee S.T. (2020). Investigation of Age-Related Changes in the Skin Microbiota of Korean Women. Microorganisms.

[57] Pouralibaba F., Babaloo Z., Pakdel F., Aghazadeh M. (2013). Serum Level of Interleukin 17 in Patients with Erosive and Non erosive Oral Lichen Planus. J. Dent. Res. Dent. Clin. Dent. Prospect..

[58] Solimani F., Pollmann R., Schmidt T., Schmidt A., Zheng X., Savai R., Muhlenbein S., Pickert J., Eubel V., Mobs C. (2019). Therapeutic Targeting of Th17/Tc17 Cells Leads to Clinical Improvement of Lichen Planus. Front. Immunol.

[59] Bain K.A., McDonald E., Moffat F., Tutino M., Castelino M., Barton A., Cavanagh J., Ijaz U.Z., Siebert S., McInnes I.B. (2020). Alopecia areata is characterized by dysregulation in systemic type 17 and type 2 cytokines, which may contribute to disease-associated psychological morbidity. Br. J. Dermatol..

[60] Kanda N., Kamata M., Tada Y., Ishikawa T., Sato S., Watanabe S. (2011). Human beta-defensin-2 enhances IFN-gamma and IL-10 production and suppresses IL-17 production in T cells. J. Leukoc. Biol..

