# Supplementary material for: Dysbiosis and Enhanced Beta-Defensin Production in Hair Follicles of Patients with Lichen Planopilaris and Frontal Fibrosing Alopecia
Source: Biomedicines. 2021 Mar 7;9(3):266. doi: 10.3390/biomedicines9030266 (PMC7999846; doi:10.3390/biomedicines9030266)
Supplement: Supplementary file 1 [file biomedicines-09-00266-s001.zip › Supplementary Figure 2.pdf]

a.

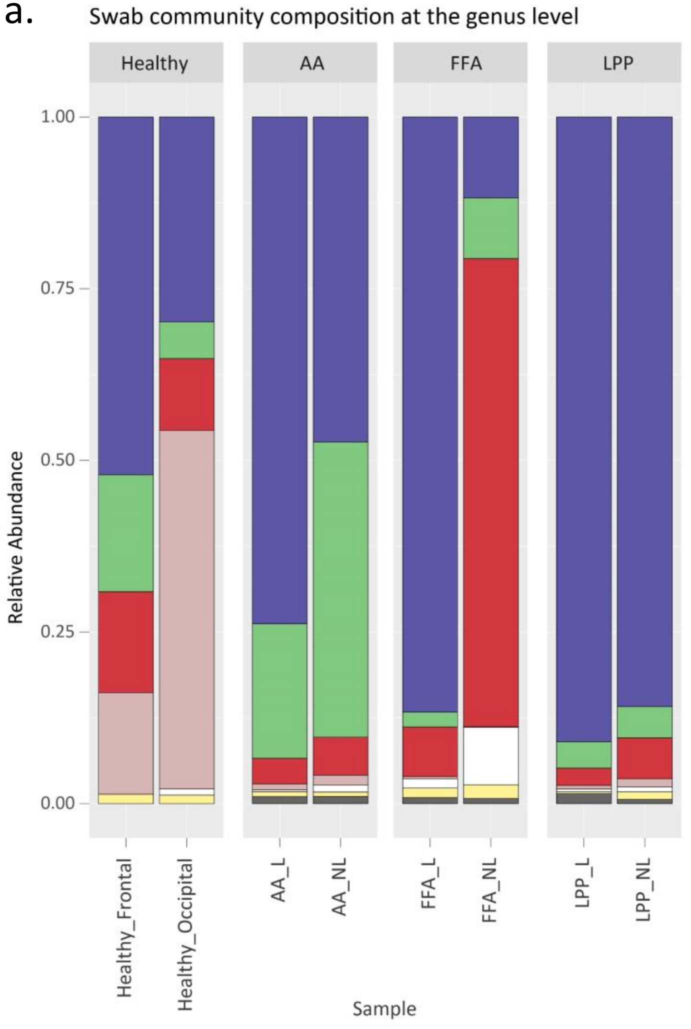

b.

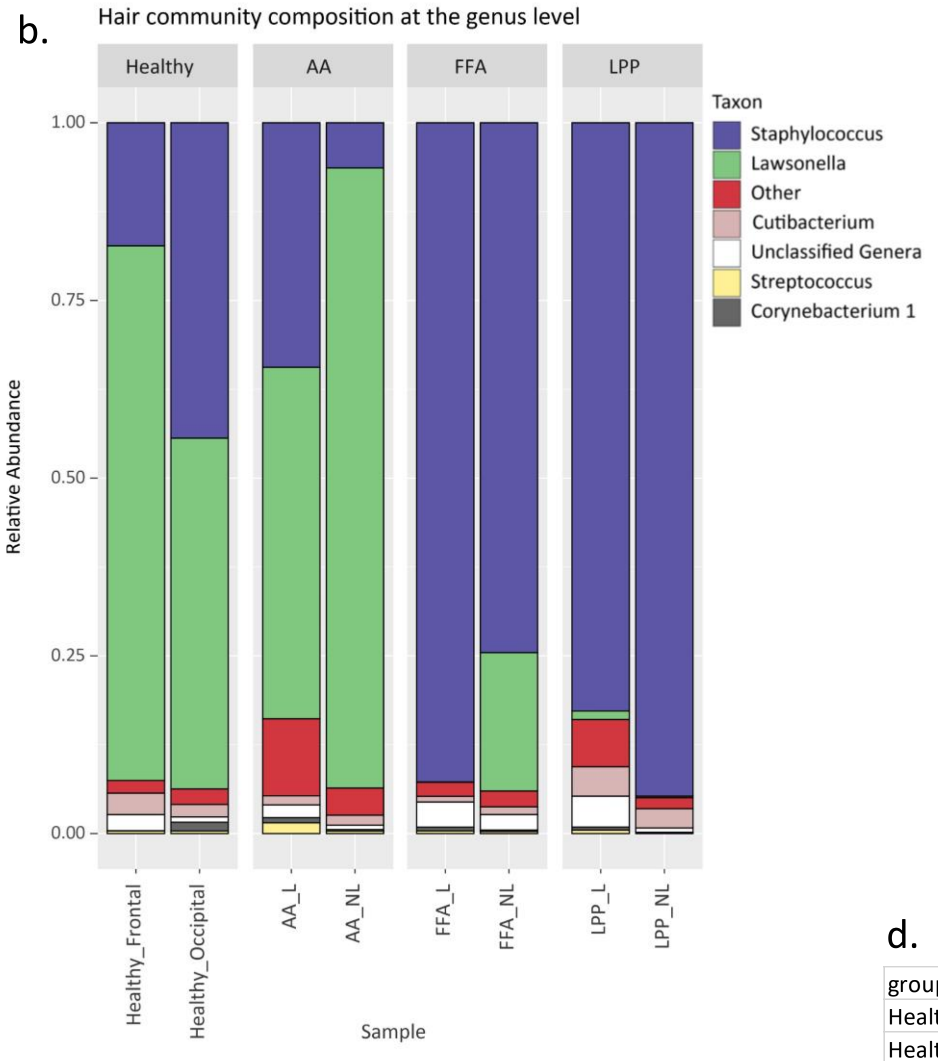

c.

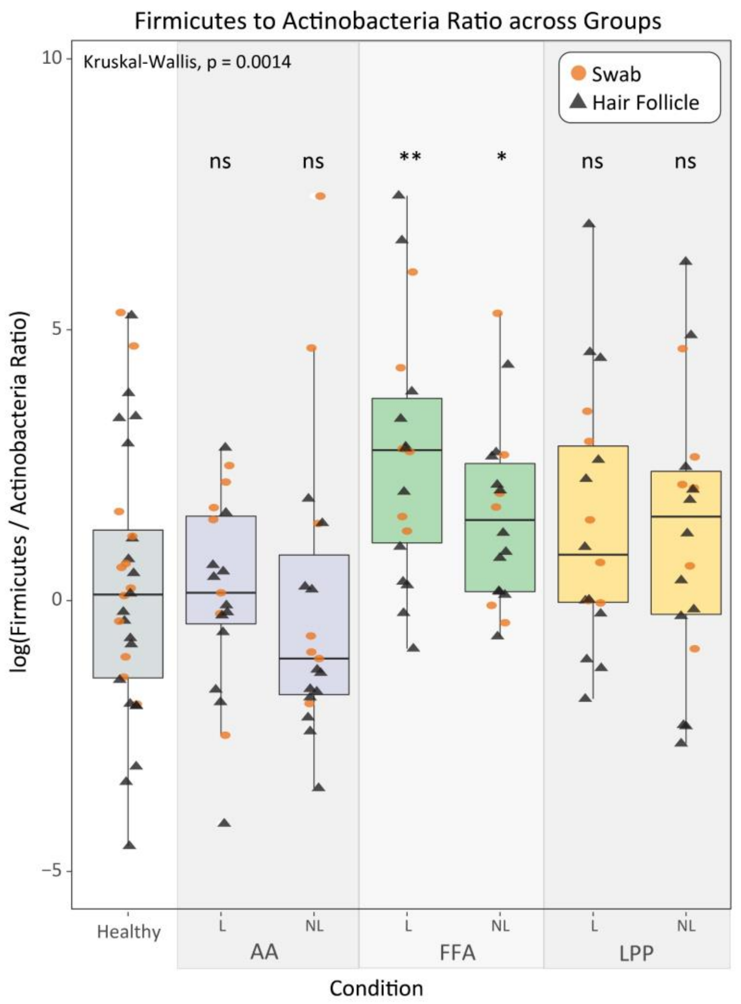

d.

group1	group2	p	p.adj	p.format	p.signif	p.adj.signi	method
Healthy	AA_L	0.455391	0.464866	0.4649	ns	ns	Wilcoxon
Healthy	AA_NL	0.464866	0.464866	0.4649	ns	ns	Wilcoxon
Healthy	FFA_L	0.000553	0.003319	0.0033	***	**	Wilcoxon
Healthy	FFA_NL	0.00954	0.028619	0.0286	**	*	Wilcoxon
Healthy	LPP_L	0.048541	0.097083	0.0971	*	ns	Wilcoxon
Healthy	LPP_NL	0.070443	0.105664	0.1057	ns	ns	Wilcoxon

Supplementary Figure 2: Preliminary data analyses excluding the male population of our control group.

Our metagenomic analyses using only female subjects, healthy (n=6) and patients AAc (n = 6), FFA (n=6), LPP (n=6). (a) Community composition of the scalp surface of healthy subjects and alopecia patients at the Genus level. Relative abundance (%) of the most abundant bacteria collected from scalp swabs, sampled from frontal (F) and occipital (O) scalp in healthy females and lesional (L) and non-lesional (NL) scalp regions in female patients. (b) Hair follicle community composition at the Genus level. Relative abundance (%) of the most abundant bacteria in hair follicles, plucked from frontal (F) and occipital (O) scalp in healthy females and lesional (L) and non-lesional (NL) scalp regions in female patients. (c) The statistical analysis suggests a trade-off between the phyla Firmicutes and Actinobacteria and the diseases under study. log(FAR) pairwise comparisons between healthy and all disease sub-groups (L for lesional and NL for non-lesional scalp sites). FAR comparisons between healthy and lesional sites show a notable increase in all diseases. In cicatricial alopecias, this difference is statistically significant (p-val<0.05). Non-lesional FFA sites also exhibit significantly increased values. Significance is based on adjusted pvalue. Orange circles and black triangles represent swab and scalp HF samples, respectively. ns indicates p-value > 0.05, * <0.05, ** < 0.01 and *** <0.001 from Mann-Whitney U-test comparing each subgroup with the reference healthy group. FAR, actinobacteria and firmicutes ratio; AA, alopecia areata circumscripta; FFA, frontal fibrosing alopecia; ns, not significant. (d) The table includes the significance (*) based on the pvalue (p) before adjustment for multiple testing and adjusted pvalue (p.adj).
