# Supplementary figures and images for: Dysbiosis and Enhanced Beta-Defensin Production in Hair Follicles of Patients with Lichen Planopilaris and Frontal Fibrosing Alopecia
Source: Biomedicines. 2021 Mar 7;9(3):266. doi: 10.3390/biomedicines9030266 (PMC7999846; doi:10.3390/biomedicines9030266)

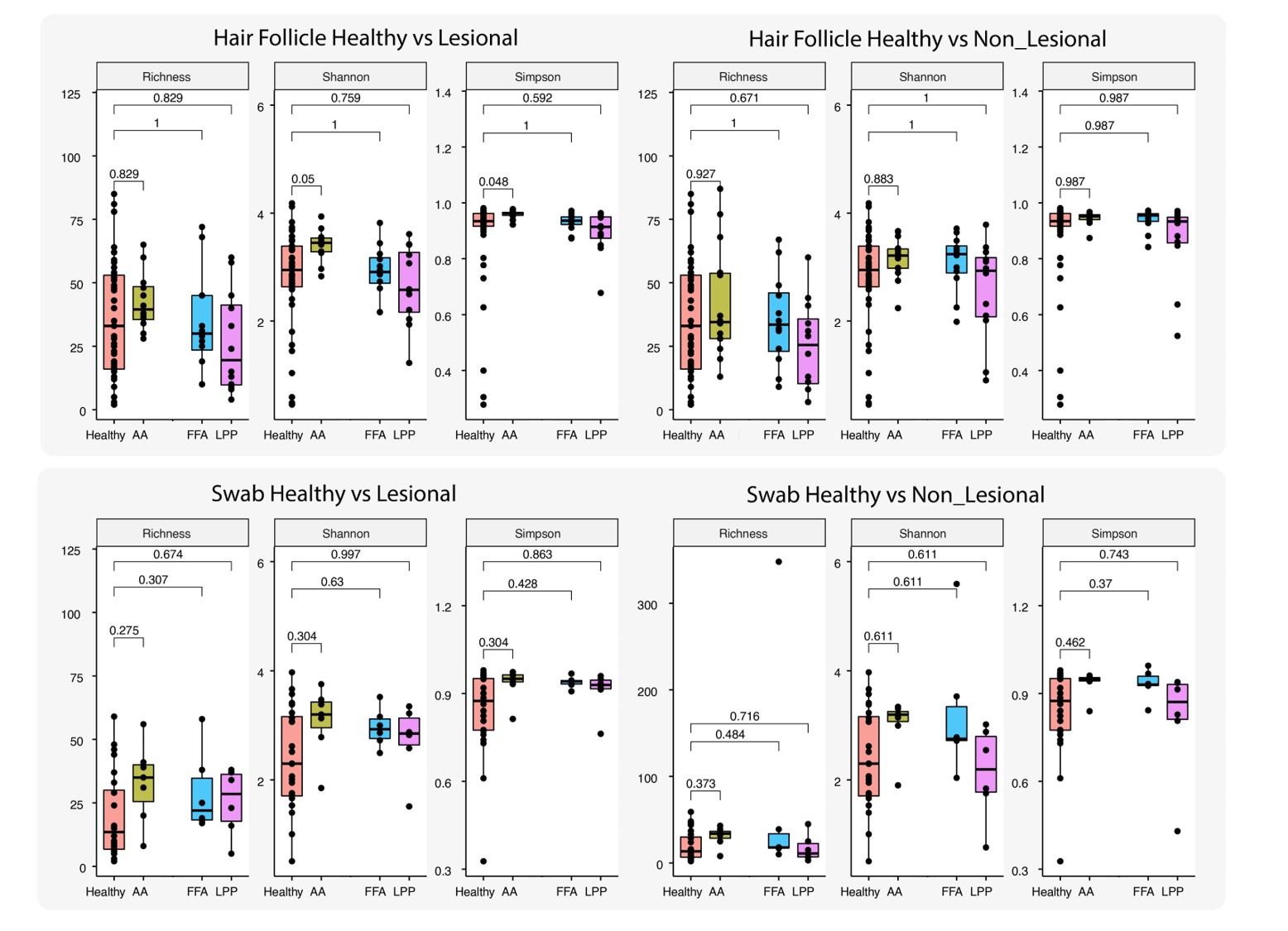

Supplement: Supplementary file 1 [file biomedicines-09-00266-s001.zip › Supplementary Figure 1.jpg]
